# Recurrent anaphylaxis highlighting methodological variability and diagnostic challenges in the basophil activation test: a case report

**DOI:** 10.1186/s40981-026-00872-6

**Published:** 2026-08-06

**Authors:** Kento Okutomi, Yuki Sugiyama, Asako Nitta, Motonobu Kimizuka, Michiaki Yamakage

**Affiliations:** Department of Anesthesiology, Sapporo Medical University School of Medicine, South 1, West 16, Sapporo, Hokkaido 060-8543 Japan

**Keywords:** Anaphylaxis, Basophil activation test, Sensitivity, Accuracy, Testing protocol

## Abstract

**Background:**

The basophil activation test (BAT) is an adjunct in identifying causative agents of anaphylaxis. However, its limited sensitivity and methodological variability may lead to inaccurate allergen identification.

**Case presentation:**

A 74-year-old woman developed anaphylaxis during anesthetic induction. BAT performed 1 week after the anaphylaxis was negative at 1-hour incubation, whereas 24-hour incubation showed slight increase in basophil activation rates with propofol (7.9%), levobupivacaine (6.1%), with cefazolin remaining negative (0.2%). At the second surgery, propofol and levobupivacaine were avoided, whereas cefazolin was re-administered, resulting in recurrent anaphylaxis. Subsequent skin testing performed 6 weeks after the second anaphylaxis was positive for cefazolin. BAT performed with a 15-minute incubation using higher drug concentrations was also positive for cefazolin (14.3%).

**Conclusions:**

Early testing after anaphylaxis, prolonged incubation, and suboptimal drug concentrations may compromise the accuracy of BAT results. This case underscores that methodological standardization and careful interpretation are essential for reliable BAT results.

**Supplementary Information:**

The online version contains supplementary material available at https://doi.org/10.1186/s40981-026-00872-6.

## Background

The basophil activation test (BAT) is an in vitro assay used to support identification of causative agents of anaphylaxis. By incubating whole blood with suspected agents and measuring basophil activation markers such as CD63 or CD203c by flow cytometry, BAT provides a minimally invasive means of allergy investigation without direct patient exposure to the suspected agent, thereby avoiding the risk of provoking recurrent anaphylaxis.

In addition to its favorable safety profile, BAT offers practical advantages. It can be performed by trained laboratory personnel in facilities equipped with flow cytometry, so the test can be completed even when clinicians are unable to perform it themselves. In contrast, skin testing must be conducted by physicians trained in allergy evaluation, including dermatologists, allergists, and anesthesiologists. This practical flexibility may be particularly useful when anesthesiologists are unable to perform skin testing due to institutional constraints or when consultation with allergists or dermatologists is not readily available.

Despite its practical advantages, skin testing remains the gold standard for identifying causative agents of anaphylaxis, with a reported sensitivity of approximately 70%, whereas the sensitivity of BAT is around 50% [1, 2]. Furthermore, testing protocols and interpretation—including reaction time, drugs and their concentrations, assay reagents, and positivity criteria—vary and are not fully standardized. Such variability may compromise the accuracy of BAT results and complicate interpretation in clinical settings.

We report a case of recurrent perioperative anaphylaxis in which BAT initially yielded a false-negative result for the true culprit drug together with slight increases in basophil activation to other suspected agents. The case underscores that BAT is a useful adjunctive tool, but one whose methodological and interpretive considerations must be recognized. Written informed consent for publication was obtained from the patient, and all procedures were conducted with appropriate consideration for patient privacy.

## Case presentation

A 74-year-old woman (height, 146 cm; weight, 59 kg) with a history of hypertension was scheduled for arthroscopic meniscal repair. She had no known allergies, and preoperative examinations were unremarkable. Surgery was attempted twice at another hospital but was aborted because of repeated anaphylaxis during anesthetic induction, and she was referred to our institution for further investigation to identify the causative agent and completion of the planned surgery.

According to the records from the referring hospital, general anesthesia for the first operation was induced with fentanyl 50 µg and propofol 120 mg and maintained with desflurane 5%. Immediately after induction, administration of cefazolin 1 g was initiated. A supraglottic airway device (SGA) was inserted, and femoral and sciatic nerve blocks were performed using 30 mL of 0.25% levobupivacaine. Eleven minutes after anesthetic induction, the administration of cefazolin was completed, and the systolic blood pressure (SBP) decreased to the 50-mmHg range and subsequently became unmeasurable. The heart rate (HR) increased to the 100–110 beats/min range. Despite administration of ephedrine (total 35 mg) and phenylephrine (total 0.3 mg), hypotension remained refractory and the surgery was aborted. The patient was awakened and the SGA was removed. She complained of chest discomfort and dyspnea, with the SpO₂ decreasing to 85%. SBP remained in the 50–60 mmHg range under a dopamine infusion of 5–7 µg/kg/min. Suspecting a cardiovascular event, transfer to an emergency hospital was arranged. Approximately 90 min after anesthetic induction, her HR dropped to 30 beats/min, and 1 mg of intravenous adrenaline was administered for resuscitation. After transfer, generalized urticaria subsequently appeared, and perioperative anaphylaxis was suspected.

One week after the first anaphylactic episode, BAT was performed at a commercial laboratory at the request of the referring hospital to identify the causative agent. Cefazolin, propofol, and levobupivacaine were tested as suspected drugs. CD203c was used as the activation marker, and incubation times of 1 h and 24 h were adopted. Each drug was evaluated at three serial dilutions (1:312.5, 1:1250, and 1:5000). The exact final drug concentrations in the reaction mixture were unavailable, as the assay was performed at the commercial laboratory. At 1 h, the negative control showed 3.8% activated basophils, and all drug-associated values were lower than the negative control at all three serial dilutions. At 24 h, the negative control showed 1.6% activated basophils. At the 1:312.5 dilution, propofol and levobupivacaine demonstrated activation rates of 7.9% and 6.1%, respectively, which were interpreted as equivocal positive results, whereas activation rates for cefazolin and for all other dilutions of propofol and levobupivacaine remained below that of the negative control (Table 1).

**Table 1:** Basophil activation test results 1 week after the first anaphylactic episode

Incubation Time [Dilution]	1 h [1:312.5]	1 h [1:1250]	1 h [1:5000]	24 h [1:312.5]	24 h [1:1250]	24 h [1:5000]
Cefazolin (10 mg/mL)	0	0	0	0.2	0	0.1
Propofol (10 mg/mL)	0.7	0	0	7.9	0	0
Levobupivacaine (7.5 mg/mL)	0	0	0	6.1	0	0
Negative control	3.8			1.6		
Positive control	61.3			37.9		

Basophil activation was assessed by CD203c expression (%) after incubation with suspected agents for 1 h and 24 h

Eight weeks after the first anaphylactic episode, the patient was scheduled for a second attempt at surgery. Because of the BAT findings, the use of propofol and levobupivacaine was avoided. General anesthesia was induced with fentanyl 50 µg and sevoflurane 5%, and an SGA was inserted. Two minutes after anesthetic induction, administration of cefazolin 1 g was initiated. Nine minutes thereafter, the SBP decreased to the 50 mmHg range and subsequently became unmeasurable. The HR increased to the 100–110 beats/min range. Wheeze was auscultated, and the SpO₂ decreased to 86%. No skin manifestations were observed. Recurrent anaphylaxis was diagnosed, and an initial 50 µg intravenous bolus of adrenaline was administered, followed by repeated additional intravenous boluses of 25–50 µg. A cumulative dose of 500 µg of intravenous adrenaline was administered within 22 min, after which a continuous adrenaline infusion was initiated at 0.1 µg/kg/min. Respiratory symptoms and hemodynamics subsequently recovered, and the surgery was aborted.

Plasma histamine and tryptase were not measured in both cases. The clinical scoring system used to assess the likelihood of anaphylaxis based on clinical symptoms [3] yielded a score of 27 (almost certain to be an immediate hypersensitivity reactions) for the first episode and 20 (very likely to be an immediate hypersensitivity) for the second episode. The patient was subsequently referred to our hospital for further allergy evaluation and surgical management.

Based on the clinical course of the two anaphylactic episodes, cefazolin was strongly suspected as the culprit drug. As surgery had already been aborted twice after induction of general anesthesia, the next operation was planned under spinal anesthesia. To identify the culprit drug and confirm the safety of the agents planned for use in the next operation, skin testing with cefazolin, lidocaine, and bupivacaine was performed at our institution 6 weeks after the second anaphylactic episode. All skin prick tests were negative, whereas intradermal testing with cefazolin (10 µg/ml) was positive, showing erythema measuring 25 × 22 mm and a wheal of 9 × 9 mm at 15 min.

Nine weeks after the second episode, BAT was repeated at our institution using the Allergenicity Kit™ (Beckman Coulter, Marseille, France). Briefly, 100 µl of heparinized whole blood was incubated with 20 µl of CRTH2-FITC / CD203c-PE / CD3-PC7 reagent and 20 µl of each suspected drug for 15 min at 37 °C. Because the total reaction volume was 140 µL, the final drug concentration in the reaction mixture was approximately 14.3% of the added drug concentration. CD203c was again used as the activation marker, but the protocol differed from the initial assay in two important respects: a 15-minute incubation time was used, and higher drug concentrations were tested. Cefazolin (10 mg/mL), propofol (10 mg/mL), and levobupivacaine (7.5 mg/mL) were each evaluated at five serial dilutions (1:10, 1:111, 1:333, 1:1000, and 1:3000) to include both the concentration used in the commercial test and the higher concentrations. BAT positivity was assessed using an activation rate ≥ 10% together with a stimulation index (SI) ≥ 2, calculated relative to the negative control. Cefazolin showed a positive response of 14.3% at the highest concentration (1:10 dilution), compared with 4.8% in the negative control, whereas propofol and levobupivacaine showed no meaningful activation across the tested concentrations (Table 2, Supplementary Figure).

**Table 2:** Basophil activation test results 9 weeks after the second anaphylactic episode

Incubation Time [Dilution]	15 min [1:10]	15 min [1:111]	15 min [1:333]	15 min [1:1000]	15 min [1:3000]
Cefazolin (10 mg/mL)	14.3	4.3	5.0	8.2	7.0
Propofol (10 mg/mL)	8.4	2.6	7.1	6.9	8.4
Levobupivacaine (7.5 mg/mL)	5.2	6.2	6.7	9.2	9.1
Negative control	4.8				
Positive control	79.8				

Basophil activation was assessed by CD203c expression (%) after a 15-minute incubation with suspected agents

At the subsequent surgery, cefazolin was avoided and clindamycin was used for antimicrobial prophylaxis. Spinal anesthesia with bupivacaine was administered, and the operation was completed uneventfully without recurrence of anaphylaxis.

## Discussion

In this case, based on the clinical scoring system, clinical course, and the results of subsequent skin testing, the diagnosis of anaphylaxis is conclusive. Perioperative anaphylaxis can be difficult to recognize, particularly when skin symptoms are not apparent at symptom onset. In the present case, the patient’s complaint of chest discomfort reasonably raised concern for a cardiovascular event. Consequently, measurement of histamine and tryptase may have been difficult during the initial episode, and adrenaline administration was delayed until approximately 90 min after the onset of anaphylaxis. In retrospect, earlier consideration of anaphylaxis as a possible diagnosis might have facilitated earlier administration of adrenaline.

After the initial episode, BAT was performed at the previous hospital owing to its practical and safety advantages. Nevertheless, the patient experienced recurrent anaphylaxis during the subsequent anesthesia. This case highlights important issues that should be addressed to optimize the clinical application of BAT in perioperative drug allergy evaluation. As was later confirmed, cefazolin was the true culprit drug, but the initial BAT yielded a false-negative result for cefazolin, whereas the slight increases in basophil activation observed with levobupivacaine and propofol did not indicate true positivity. Although these slight increases were scientifically regarded as non‑specific reactions, they were interpreted as possibly positive and therefore functioned as false‑positive results in the clinical context. When the results are uncertain like this initial BAT, repeat testing or consultation with an allergy specialist may be appropriate. These misleading findings contributed to re-exposure to cefazolin and recurrence of anaphylaxis.

The false-negative cefazolin result in the initial BAT may be explained, at least in part, by the timing of testing and the concentration of the drug used. First, the initial BAT was performed 1 week after the anaphylactic episode. In skin testing, evaluation is generally recommended 4–6 weeks after anaphylaxis because transient post-event hyporesponsiveness may reduce cellular reactivity, and a recovery period is therefore advised to restore cellular responsiveness [4]. Although the optimal timing of BAT after anaphylaxis has not been clearly established, basophils may exhibit a similar transient hyporesponsive state. In the present case, early testing could therefore have contributed to the false-negative result.

Second, the cefazolin concentration used in the initial BAT may also have been insufficient. Several studies have examined the diagnostic performance of BAT for β-lactam antibiotics [5, 6]. These reports indicate that BAT responses can be dependent on the concentrations applied. Importantly, cefazolin in particular has been reported to require relatively high concentrations to elicit a detectable in vitro response in a concentration-dependent manner (approximately ≥ 0.6 mg/mL up to 10 mg/mL) [7]. The second BAT, performed nine weeks after the anaphylactic episode and after cefazolin had already been identified as the culprit drug by skin testing, provided an opportunity to examine whether modification of the BAT protocol, including the use of a higher cefazolin concentration, would alter the BAT outcome. Accordingly, the second BAT included both the concentration used in the initial BAT and a higher concentration (1 mg/mL), which falls within the previously reported range and corresponds to a 1:10 dilution of the clinical preparation. This approach yielded a clearly positive response, suggesting that the cefazolin concentration used in the initial BAT may have been insufficient to trigger detectable basophil activation. Although enhanced sensitization after the recurrent anaphylaxis was likely present in the second BAT, the early timing and the potentially low cefazolin concentration in the initial BAT may both have contributed to the false-negative result. When BAT is performed for diagnostic purposes before the culprit drug has been identified, testing at higher cefazolin concentrations may be considered.

In addition to the factors contributing to the false‑negative result, the initial BAT also showed slight non-specific increases in basophil activation with levobupivacaine and propofol, which were interpreted as possibly positive at the time and therefore warrant separate consideration. These findings may be explained by two main factors: the incubation time with the suspected agents and the diagnostic threshold used to define positivity. First, with regard to incubation time, a duration of 15–30 min is commonly recommended for BAT, whereas the initial BAT in this case used prolonged incubation periods of 1 h and 24 h [4, 8, 9]. Although a 1-hour incubation may still be considered acceptable, 24 h is considerably longer than typically recommended. Prolonged incubation may impair cellular viability and alter responsiveness to stimuli. Consistent with this concern, CD203c expression in the positive control decreased from 61.3% at 1 h to 37.9% at 24 h, indicating altered cellular responsiveness with prolonged incubation. Furthermore, such prolonged incubation is unlikely to be necessary given the underlying mechanism of anaphylaxis.

Second, the criteria for positivity applied in BAT may also have influenced the results. BAT positivity is commonly assessed using either predefined activation thresholds (for example, ≥ 10% basophil activation in at least two consecutive tests) or the SI, calculated relative to the negative control, in which the activation marker expression is typically < 5% [10, 11]. An SI > 2 is widely used because of its simplicity [12]. However, SI‑based interpretation may misclassify nonspecific activation as positive and is strongly affected by the activation rate in the negative control, particularly when this value is very low. In the present case, the basophil activation rate in the negative control was 1.6% at the 24‑hour incubation time, meaning that even modest nonspecific activation could easily yield an SI > 2. The basophil activation rates observed for levobupivacaine (6.1%) and propofol (7.9%) at 24 h were relatively low. If a predefined activation threshold such as ≥ 10% basophil activation had been applied, these results would have been interpreted as negative. Incorporating both SI and an absolute activation threshold may therefore help improve the accuracy of BAT interpretation.

The two BATs differed in multiple methodological aspects, including timing, drug concentrations, incubation time, and may also have differed in testing kits, laboratories, and other procedural details. Because the initial BAT was outsourced to a commercial laboratory, the details of the testing procedure were limited to the information provided in the referral document. Differences in test kits, laboratories, and procedural nuances are challenges inherent to many diagnostic assays, not only BAT. To improve the reliability of outsourced BAT, it would be desirable to establish a process in which essential testing parameters—such as incubation time and drug concentrations—can be specified in advance, together with broader standardization of incubation conditions, drug concentrations, and positivity criteria in future practice.

Finally, whether using BAT or skin testing, positive reactions to multiple suspected agents should be interpreted with caution. Epidemiological studies and large perioperative anaphylaxis registries indicate that—excluding cross-reactivity—it is exceedingly rare for more than one drug to represent the true causative agent of anaphylaxis [13, 14]. Even for relatively common triggers, such as antibiotics, rocuronium, or sugammadex, the reported incidence is approximately 1 in 20,000 exposures [15]. From a probabilistic perspective, therefore, the likelihood that multiple unrelated agents represent true positives in a single patient is extremely low.

Accordingly, when multiple positive results are obtained—particularly when the responses are equivocal—repeat or alternative testing should be considered. In the present case, slight activation to levobupivacaine and propofol was overinterpreted as positive. A more cautious interpretation, taking into account both epidemiological probability and test variability, would help minimize the risk of misclassification in such situations.

In conclusion, this case demonstrates that a false-negative BAT result, combined with a non-specific increase that was misinterpreted as possibly positive, can contribute to recurrent anaphylaxis. Careful interpretation of BAT results, including attention to testing conditions and epidemiological probability, is therefore essential. Notably, the second BAT, performed after an appropriate interval and with optimized drug concentrations, yielded accurate results, indicating that BAT remains a convenient, minimally invasive, and potentially valuable adjunctive diagnostic tool. Further accumulation of cases and standardization of incubation conditions, drug concentrations, and positivity criteria will be important for improving its diagnostic reliability in perioperative anaphylaxis.

## Supplementary Information

Supplementary Material 1.

## Data Availability

Not applicable due to patient privacy concerns.

## Abbreviations

BAT: basophil activation test
SGA: supraglottic airway device
SBP: systolic blood pressure
HR: heart rate
SI: stimulation index

## References

[1] Hoffmann HJ, Santos AF, Mayorga C, Nopp A, Eberlein B, Ferrer M, et al. The clinical utility of basophil activation testing in diagnosis and monitoring of allergic disease. Allergy. 2015;70(11):1393–405. 10.1111/all.12698 26198455

[2] Orihara M, Takazawa T, Nagumo K, Sakamoto S, Horiuchi T, Saito S. Interpreting the results of early skin tests after perioperative anaphylaxis requires special attention: a case report and review of literature. J Anesth. 2020;34(4):624–9. 10.1007/s00540-020-02802-x PMC7378110 32476042

[3] Hopkins PM, Cooke PJ, Clarke RC, Guttormsen AB, Platt PR, Dewachter P, et al. Consensus clinical scoring for suspected perioperative immediate hypersensitivity reactions, Br J Anaesth. 2019;123(1):e29–37. 10.1016/j.bja.2019.02.029 31029409

[4] Chahine C, Zidane Z, Mohtadi K, Souat S, El Azhary K, Saïle R, et al. Diagnosis of propofol allergy (the innovative role of the basophil activation test in the identification of allergen): a case report. Pan Afr Med J. 2025;50:82. 10.11604/pamj.2025.50.82.46400 PMC12271875 40687171

[5] Sanz ML, Gamboa PM, Antepara I, Uasuf C, Vila L, Garcia-Aviles C, et al. Flow cytometric basophil activation test by detection of CD63 expression in patients with immediate-type reactions to betalactam antibiotics. Clin Exp Allergy. 2002;32(2):277–86. 10.1046/j.1365-2222.2002.01305.x 11929494

[6] Abuaf N, Rostane H, Rajoely B, Gaouar H, Autegarden JE, Leynadier F, et al. Comparison of two basophil activation markers CD63 and CD203c in the diagnosis of amoxicillin allergy. Clin Exp Allergy. 2008;38(6):921–8. 10.1111/j.1365-2222.2008.02960.x 18331364

[7] Horiuchi T, Takazawa T, Orihara M, Sakamoto S, Yokohama A, Takahashi J, et al. Required cefazolin concentration to maximize diagnostic accuracy of the basophil activation test for cefazolin-induced anaphylaxis. J Anesth. 2018;32(6):797–805. 10.1007/s00540-018-2555-z 30225721

[8] Sturm EM, Kranzelbinder B, Heinemann A, Groselj-Strele A, Aberer W, Sturm GJ. CD203c-based basophil activation test in allergy diagnosis: characteristics and differences to CD63 upregulation. Cytometry B Clin Cytom. 2010;78(5):308–18. 10.1002/cyto.b.20526 20533392

[9] Chirumbolo S, Vella A, Ortolani R, De Gironcoli M, Solero P, Tridente G, et al. Differential response of human basophil activation markers: a multi-parameter flow cytometry approach. Clin Mol Allergy. 2008;6:12. 10.1186/1476-7961-6-12 PMC2584049 18925959

[10] Monneret G, Benoit Y, Debard AL, Gutowski MC, Topenot I, Bienvenu J. Monitoring of basophil activation using CD63 and CCR3 in allergy to muscle relaxant drugs. Clin Immunol. 2002;102(2):192–9. 10.1006/clim.2001.5156 11846462

[11] Sudheer PS, Hall JE, Read GF, Rowbottom AW, Williams PE. Flow cytometric investigation of peri-anaesthetic anaphylaxis using CD63 and CD203c. Anaesthesia. 2005;60(3):251–6. 10.1111/j.1365-2044.2004.04086.x 15710010

[12] Orihara M, Takazawa T, Horiuchi T, Sakamoto S, Nagumo K, Tomita Y, et al. Comparison of incidence of anaphylaxis between sugammadex and neostigmine: a retrospective multicentre observational study. Br J Anaesth. 2020;124(2):154–63. 10.1016/j.bja.2019.10.016 31791621

[13] Harper NJN, Cook TM, Garcez T, Farmer L, Floss K, Marinho S, et al. Anaesthesia, surgery, and life-threatening allergic reactions: epidemiology and clinical features of perioperative anaphylaxis in the 6th National Audit Project (NAP6). Br J Anaesth. 2018;121(1):159–71. 10.1016/j.bja.2018.04.014 29935567

[14] Li J, Best OG, Rose MA, Green SL, Fulton RB, Capon MJ, et al. Assessing cross-reactivity to neuromuscular blocking agents by skin and basophil activation tests in patients with neuromuscular blocking agent anaphylaxis. Br J Anaesth. 2019;123(1):e144–50. 10.1016/j.bja.2019.03.001 30961915

[15] Takazawa T, Horiuchi T, Nagumo K, Sugiyama Y, Akune T, Amano Y, et al. The Japanese Epidemiologic Study for Perioperative Anaphylaxis, a prospective nationwide study: allergen exposure, epidemiology, and diagnosis of anaphylaxis during general anaesthesia. Br J Anaesth. 2023;131(1):159–69. 10.1016/j.bja.2023.02.018 36990827

